# Effects of 3 months of short sessions of controlled whole body vibrations on the risk of falls among nursing home residents

**DOI:** 10.1186/1471-2318-13-42

**Published:** 2013-05-06

**Authors:** Charlotte Beaudart, Didier Maquet, Mélanie Mannarino, Fanny Buckinx, Marie Demonceau, Jean-Michel Crielaard, Jean-Yves Reginster, Olivier Bruyère

**Affiliations:** 1 Department of Public Health, Epidemiology and Health Economics, University of Liège, CHU – Bât. B.23, Liège, 4000, Belgium; 2 Department of Motricity Sciences, University of Liège, Liège, Belgium

**Keywords:** Whole-body-vibration, Nursing home, Falls

## Abstract

**Background:**

Fatigue, lack of motivation and low compliance can be observed in nursing home residents during the practice of physical activity. Because exercises should not be too vigorous, whole body vibration could potentially be an effective alternative. The objective of this randomized controlled trial was to assess the impact of 3-month training by whole body vibration on the risk of falls among nursing home residents.

**Methods:**

Patients were randomized into two groups: the whole body vibration group which received 3 training sessions every week composed of 5 series of only 15 seconds of vibrations at 30 Hz frequency and a control group with normal daily life for the whole study period. The impact of this training on the risk of falls was assessed blindly by three tests: the Tinetti Test, the Timed Up and Go test and a quantitative evaluation of a 10-second walk performed with a tri-axial accelerometer.

**Results:**

62 subjects (47 women and 15 men; mean age 83.2 ± 7.99 years) were recruited for the study. No significant change in the studied parameters was observed between the treated (n=31) and the control group (n=31) after 3 months of training by controlled whole-body-vibrations. Actually, the Tinetti test increased of + 0.93 ± 3.14 points in the treated group against + 0.88 ± 2.33 points in the control group (p = 0.89 when adjusted). The Timed Up and Go test showed a median evolution of - 1.14 (− 4.75-3.73) seconds in the treated group against + 0.41 (− 3.57- 2.41) seconds in the control group (p = 0.06). For the quantitative evaluation of the walk, no significant change was observed between the treated and the control group in single task as well as in dual task conditions.

**Conclusions:**

The whole body vibration training performed with the exposition settings such as those used in this research was feasible but seems to have no impact on the risk of falls among nursing home residents. Further investigations, in which, for example, the exposure parameters would be changed, seem necessary.

**Trial registration:**

Trial registration number: NCT01759680

## Background

Falls are a leading cause of morbidity and mortality in older people. At least 30% of people aged 65 years and older fall every year and this incidence increases in institutionalized people [1]. Consequences of this important public health issue may be serious: post-fall syndrome, dependency, hospitalisation, increasing of mortality, significant risk of recurrence and so on [2]. The age-related decreases in postural control and muscle strength have been identified as major risk factors for falls [3]. Therefore, an intervention to prevent these conditions could potentially reduce the frequency of falls. Numerous studies have demonstrated the beneficial effects of physical exercise on falls [4-6]. Moreover, a recent meta-analysis showed that physical activity prevents the risk of falls with an odds ratio of 0.84 (95% CI : 0.77-0.91) [7]. This meta-analysis seems to state that a high dose of physical activity produces more important results than a lower dose. Besides, to avoid the routine practice of physical activity and because too vigorous exercises could be difficult for some nursing home residents, whole body vibration (WBV) could potentially be an effective alternative. However, because of the many exclusion criteria to whole body vibration, this therapy is not accessible to everyone. It is also important to add that the balance risk-benefits of the therapy has not been clearly established and is still discussed in the literature [8].

In whole body vibration, exercises are performed on a platform that generates vertical sinusoidal vibrations to produce a stimulation of the muscle spindles and induce muscles contractions [9]. Clinical studies suggest that WBV may improve muscular performance and body balance in the elderly [9-17]. Those studies used either a Power-Plate^®^ or a Galileo^®^ device. Another vibration device, the Vibrosphere^®^ is characterized by a spherical base. In addition to the standard muscle stimulation, the Vibrosphere^®^, thanks to its spherical form, could activate various sensors (visual, vestibular, cutaneous) and thus produce an additional beneficial effect on balance, a major factor reducing the risk of falls.

Therefore, this study investigates, as primary objective, the effects of the use of a Vibrosphere^®^ on the risk of falls among the elderly. We performed a randomized controlled study with the hypothesis of a reduction of the risk of falls in the treated group compared to the control group. We also defined two secondary objectives that consist to assess the impact of the training on the number of falls incurred by the patients and to assess the prediction capacity of response to Vibrosphere^®^ according to baseline characteristics.

In the literature, a relatively high rate of dropout in WBV groups is observed. Indeed, in most studies, more than 20% of patients from the treated group interrupted the study before its end. Our hypothesis is that the exposures used in the different studies are a little too vigorous for elderly people. Therefore, we decided to expose our population to a smaller vibration period than usually observed in other studies to analyse if such a protocol could be more suitable for patients.

## Methods

### Participants

Patients were recruited from two nursing homes in Liège, Belgium. Exclusion criteria were patients 1) weighing more than 150 kg, 2) having electronic implants (e.g. pacemaker, brain stimulators), 3) having prosthetic hips or knees, 4) suffering from epilepsy, bleeding disorders, inflammatory abdominal disorders or at high risk of thromboembolism.

Patients were randomly assigned to the WBV group or to the control group. We performed the randomisation by blocks of four with a computer-generated randomisation procedure. An identification number and a randomisation number were created for each participant.

To ensure sufficient statistical power, a preliminary calculation of the number of patients needed for the study was performed. This calculation was based on a previous study [12] that showed a significant increase of 5.6 points in the Tinetti test for the treated group and a decrease of 0.3 points for the control group, which represent a difference of 5.9 points between groups. Based on this difference, an α value of 0.05 and a power of 90%, it appeared that 46 patients would be required. Assuming a dropout rate of about 8% [18], the study sample had to consist of at least 50 patients, 25 patients in the WBV group and 25 in the control group.

The study was approved by the “Comité d'Ethique Hospitalo-Facultaire Universitaire de Liège ». All participants gave written informed consent.

### Whole body vibration intervention

**Figure 1:**
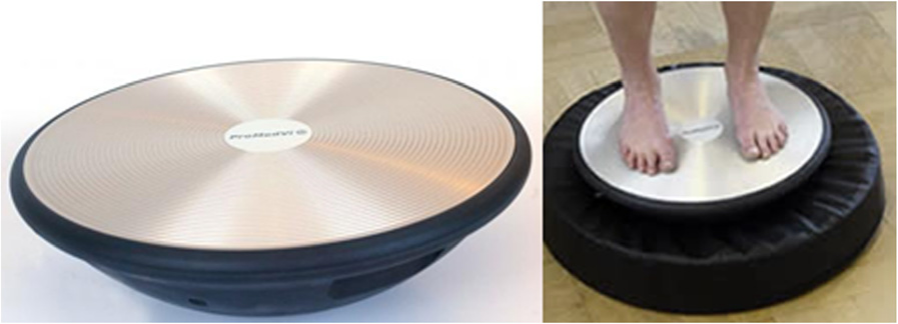
The Vibrosphere^®^ device.

The WBV group performed exercises three times a week during three months on a sinusoidal vibration platform (Vibrosphere^®^, Figure 1). Exercises consisted of standing up, shoes removed, in a bipodal station with a knee flexion (as if skiing, no specified angle) on this vertical vibration platform for 5 series of 15 seconds of vibrations at 30 Hz intensity, 2mm of amplitude, alternating with 30 seconds of rest. As the vibration device has a spherical base, four different cushions with various density and thickness can be placed under the platform to decrease more or less the difficulty. Given the physical health of our population, we decided to place the cushion with the lowest density and the highest thickness (10 cm) to facilitate the training as much as possible. The device was placed in front of wall-bars to reassure patients in case of imbalance. Patients were asked not to hold onto these wall-bars during the training but could place their hand close to them.

Four persons supervised the trainings: 2 physiotherapists and the first and third authors of this article. Once again, because of the spherical base of the device, supervision was necessary. Each training session was supervised by one of the supervisors. Some patients came to the training room by themselves, but for most of them, supervisors had to pick them up from their room to the training room.

Patients, in the control group, were requested neither to change their lifestyle during the study nor to get involved in any new type of physical activity.

### Outcome measurement

Risk of falls for each patient was assessed blindly. Thanks to the two locations, the investigators who conducted the assessments in one nursing home supervised patients from the other nursing home so that they would not know to which group the patient, they were assessing, belonged. The assessments consisted of 3 tests at baseline and after 3 months.

#### Tinetti test

The Tinetti test was used to assess the balance and gait abnormalities of the patients. With a specificity of 80% and a sensibility of 74% [19], it is one of the most widely used tests in this field. It consists of two subtests: a balance test (9 items scored on 16 points) and a gait test (7 items scored on 12 points). According to Tinetti [20], a total score of less than 19 points indicates severe risk of falls, a score between 19 and 24 points indicates moderate risk of falls and a score of more than 24 points indicates low risk of falls.

#### Timed Up and Go test (TUG)

The Timed up and Go test (which is a modified version of the Get Up and Go test [21]) was used to assess the functional mobility of patients. With a specificity of 87% and a sensibility of 87%, this test reflects both dynamic body balance and muscle power [22].

For the TUG test patients are asked to rise from a standard armchair, walk to a marker 3 meters away, turn, walk back and sit down again. A time of more than 14 seconds suggests a high risk of falls [22].

#### Quantitative walking analysis (Locometrix^®^)

This test consists of a quantitative evaluation of a 10-second walk performed with the Locometrix^®^ system, a validated instrument for elderly fallers [23]. Patients have to walk 3 times a distance of 20 meters with a sensor, which collects the accelerations measured at a frequency of 100 Hertz (Hz), placed around the waist by means of a semi-elastic belt. The gait variables analysed are as follow:

1) Speed during the walking test (expressed in meters per second) was measured using a chronometer;

2) The stride frequency or number of cycles per second (expressed in Hertz) was calculated from the cranio-caudal acceleration following application of a Fourier transform;

3) The stride length (expressed in meters) was obtained in meters by dividing walking speed by cycle frequency;

4) The stride regularity and the stride symmetry (expressed in arbitrary units) are both measures of the similarity (in terms of duration and amplitude) of the shape of cranio-caudal acceleration curves. The regularity compares one step to another and the symmetry right to left strides;

5) The mechanical power cranio-caudal (expressed in Watts per kilogram) measures the subject's state of kinesis. This power defines the vertical movements experienced by the centre of gravity when walking;

6) The mechanical power anteroposterior and mediolateral (expressed in Watts per kilogram) quantify the state of kinesis in these two areas as well as antero-posterior and lateral dynamic instabilities.

Those parameters were assessed during a sample of 10 second of stabilized walk recorded during each 20-meter distance.

This test is an evaluation criterion of the risk of falls because some of its measures are correlated with the risk of falls of elderly patients. For example, a gait speed less than 0.56 m/s is considered as a risk factor for falls [24].

Locometrix^®^ test was performed only with patients who did not use walking assistance either during the baseline evaluation or during the 3-month evaluation.

### Falls

Falls were recorded by the nurses in the nursing homes. Falls were defined as “unintentionally coming to rest on the ground, floor, or other lower level.” Nurses completed the fall record with the date, time, and circumstances of the falls. They also noted possible consequences of falls.

### Statistical analysis

A Shapiro-Wilk test verified the normal distribution for all parameters. When data were normally distributed, a student’s t-test was used to assess differences between the two groups or differences within groups. Non-parametric statistics were used when data were not normally distributed (Man Whitney U test: between groups differences and Wilcoxon test: within-groups differences). For qualitative variables, a Chi^2^ of Pearson was performed.

Quantitative variables that were normally distributed were expressed as mean ± standard deviation (SD) and quantitative variables that were not normally distributed were expressed as median (percentile 25, percentile 75). Qualitative variables were reported as absolute and relative frequencies (%).

Analyses were adjusted for baseline variables that were significantly different between WBV and control groups by means of a multiple regression.

A logistic regression was realized to assess the response prediction to the Vibrosphere^®^ according to patients’ baseline characteristics.

Results were considered statistically significant when 2-tailed p values were less than 0.05.

Analyses were executed with the software Statistica 9.1.

Data were analysed on an intention-to-treat basis. Data of dropouts who returned for follow-up measurements were also included in the analysis.

## Results

### Demographics and baseline characteristics

Sixty-two patients (47 women and 15 men aged 83.2 ± 7.99 years) were recruited for the study. Half of them (31) were randomized to the WBV group and the other 31 patients to the control group. Baseline characteristics of the two groups are summarized in Table 1.

**Table 1:** Demographic data of subjects in both WBV and control groups

**Parameter**	**n**	**WBV**	**n**	**Control**	**p-value**
Sex					
Women	31	20 (64.5)	31	27 (87.1)	.04
Age (years)	31	82.2 ± 9.02	31	84.2 ± 6.83	.31
Height (cm)	31	163 (159–175)	31	162 (158–166)	.20
Weight (kg)	31	68.8 ± 14.1	31	58.9 ± 12.6	<.01
Body Mass Index (kg/m^2^)	31	25.2 ± 4.13	31	22.5 ± 3.91	.01
Glasses					
Yes	31	24 (77.4)	31	26 (83.9)	.52
Walking assistance					
Yes	31	17 (54.8)	31	12 (38.7)	.20
Physiotherapy					
Yes	31	13 (41.9)	31	8 (25.8)	.18
Medication (number)	31	8.58 ± 3.41	30	8.90 (4.01)	.74
Medication					
>4	31	30 (96.8)	30	27 (90.0)	.29
Comorbidities (number)	31	3.00 (2.00-5.00)	31	3.00 (2.00-4.00)	.42
Mini-Mental State Examination (score /30)	30	26.0 (19.0-28.0)	29	23.0 (15.0-27.0)	.04
History of falls within 6 months					
Yes	31	11 (35.5)	31	10 (32.3)	.79
History of fractures					
Yes	31	7 (22.6)	31	6 (19.4)	.75

The two groups of patients did not differ significantly except for three characteristics: there were more women in the control group than in the WBV group (p= 0.04), patients in the control group presented lower Body Mass Index than patients in the WBV group (p< 0.01) and patients in the control group presented lower Mini Mental State Examination than patients in the WBV group (p= 0.04).

Regarding the risk of fall (Table 2), the total Tinetti test score at baseline raised at 22.9 ± 3.99 points in the treated group and 22.2 ± 4.25 in the control group. TUG test median was 19.0 (14.6-27.9) seconds in the WBV group and 19.1 (13.2-26.6) seconds in the control group.

**Table 2:** – Baseline data of subjects in both WBV and control groups

**Parameter**	**n**	**WBV**	**n**	**Control**	**p-value**
**Tinetti Test**					
Balance (score /16)	31	13.6 ± 2.06	30	12.9 ± 2.63	.26
Gait (score /12)	31	9.35 ± 2.30		9.27 ± 2.23	.88
Tinetti total (score /28)	31	22.9 ± 3.99		22.2 ± 4.25	.47
**Timed Up and Go test** (seconds)	31	19.0 (14.6-27.9)	30	19.1 (13.2-26.6)	.76
**Locometrix^®^ test**					
Gait speed (meter/second)					
Simple task	13	0.90 ± 0.21	14	0.93 ± 0.24	.77
Dual task	10	0.70 ± 0.23	12	0.76 ± 0.21	.51
P-Value		.01		<.01	
Stride frequency (cycle/second)					
Simple task	13	1.05 ± 0.20	14	1.07 ± 0.26	.82
Dual task	10	0.82 ± 0.30	12	0.89 ± 0.23	.56
P-Value		.07		.08	
Stride length (meter)					
Simple task	13	0.86 ± 0.10	14	0.88 ± 0.18	.65
Dual task	10	0.89 ± 0.20	12	0.86 ± 0.10	.63
P-Value		.65		.95	
Stride symmetry (arb. unit.)					
Simple task	13	199.6 ± 51.3	14	183.6 ± 55.2	.44
Dual task	10	159.5 ± 66.8	12	194.9 ± 38.9	.14
P-Value		.24		.57	
Stride regularity (arb. unit.)					
Simple task	13	200.6 ± 41.1	14	206.0 ± 64.0	.80
Dual task	10	149.4 ± 77.1	12	143.8 ± 68.4	.86
P-Value		.03		<.01	
Cranio-caudal mechanic power					
(Watts/kg)	13	0.70 (0.58-1.20)	14	1.00 (0.63-2.36)	.62
Simple task	10	0.63 (0.40-1.09)	12	0.66 (0.38-1.23)	.77
Dual task		.01		.01	
P-Value					
Antero-posterior mechanic power					
(Watts/kg)	13	0.47 (0.35-0.83)	14	0.58 (0.31-0.69)	.94
Simple task		0.44 (0.29-0.78)		0.38 (0.29-0.54)	
Dual task		.33		.05	
P-Value	10		12		.77
Medio-lateral mechanic power					
(Watts/kg)	13	0.45 (0.25-0.55)	14	0.39 (0.29-0.96)	.65
Simple task	10	0.41 (0.22-0.72)	12	0.38 (0.23-0.72)	.97
Dual task		.21		.08	
P-Value					
Counting speed (number/second)					
Simple task	10	0.89 ± 0.84	12	1.01 ± 0.22	.24
Dual task	10	0.69 ± 0.30	12	0.76 ± 0.28	.55
P-Value		<.01		<.01	

*WBV* = Whole Body Vibration.

### Dropout and compliance

During the study 6 subjects (19.4%) dropped out of the WBV group: 4 for medical reason (2 hip pains who seemed to be related to the training, 1 fracture of the malleolus unrelated to the study and 1 hospitalisation for total hip replacement unrelated to the study) and 2 for failure to meet the inclusion criteria. Although those 6 patients did not complete the study, a post-evaluation was performed on five of them in an intention-to-treat analysis. It was impossible to perform this evaluation on the sixth patient because he died in hospital from a nosocomial infection. A second death, from gastrointestinal bleeding, was recorded during the 3-month study in the WBV group, but the post-evaluation had already been made for this patient since he left the study one month before dying.

**Figure 2:**
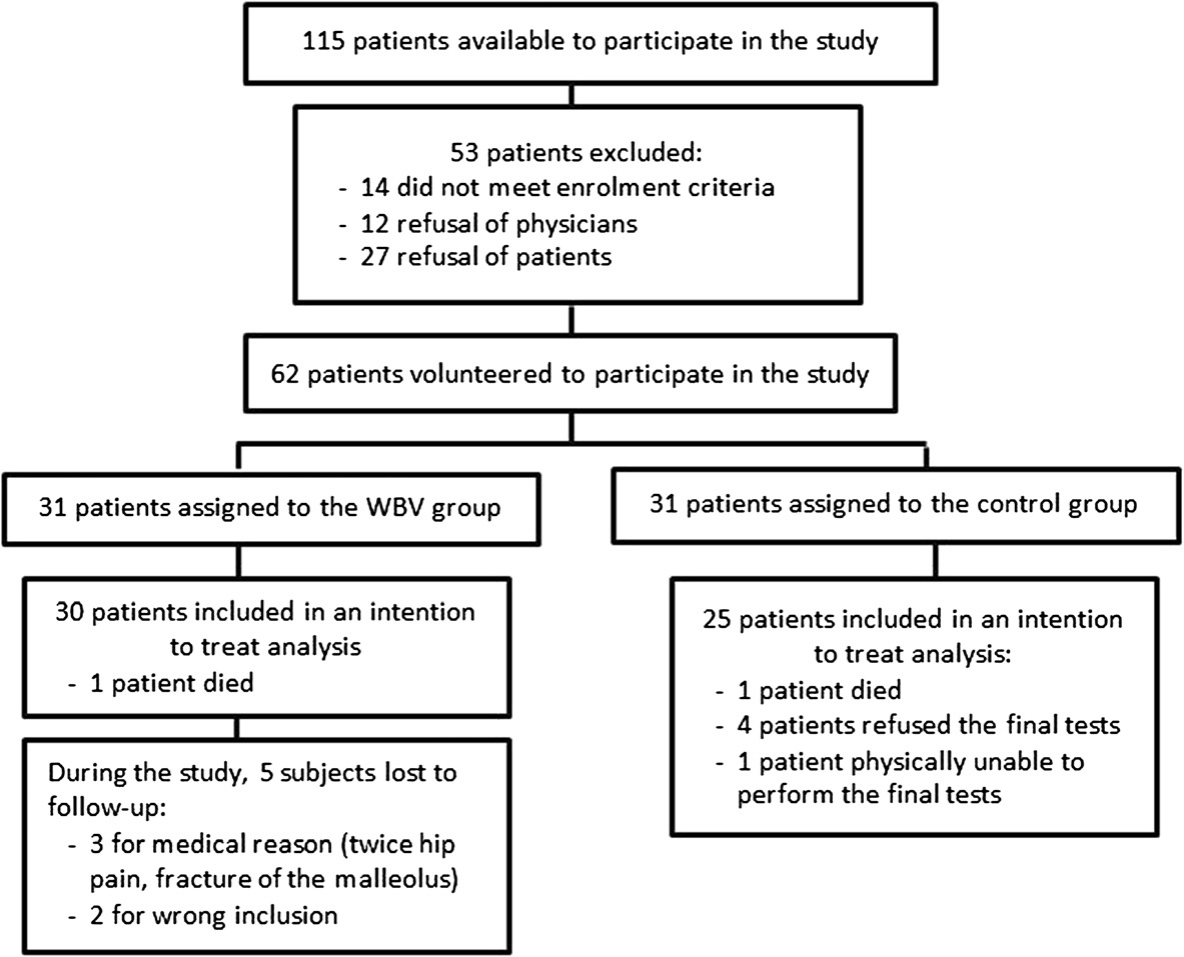
Flowchart of the study.

In the control group, 25 patients completed the final test. Actually, one patient died during the study from cardiac decompensation followed by acute pulmonary oedema, 4 patients refused to perform the post-evaluation and 1 patient was unable to complete the post-tests (Figure 2).

To summarize, although the same number of patients dropped out of both groups and did not complete the study, post-evaluations were performed in 96.7% of the patients in the WBV group and in 80.6% of the patients in the control group.

Regarding the compliance, we counted that 91.9% of the exercise sessions were performed. Unattended sessions are explained by various health conditions (gastroenteritis epidemic, influenza, bronchitis and fatigue), some travels and one hospitalization for prostatectomy.

### Tinetti test

In the WBV group, we observed a balance increase of + 0.07 ± 1.91 point and a gait increase of + 0.87 ± 1.91 point after 3 months which represent a total Tinetti increase of + 0.93 ± 3.14 point. In the control group, an increase was also observed for balance and gait parameters with + 0.40 ± 2.10 and + 0.48 ± 1.64 points respectively, which represent a total Tinetti increase of + 0.88 ± 2.33 point (Table 3). Intergroup difference is not significant (p= 0.89 when adjusted for sex, Body Mass Index and Mini Mental State Examination).

**Table 3:** Evolution of the Tinetti test and the Timed Up and Go test for both groups

	**WBV (n = 30)**	**Control (n = 25)**	**p-value**	p-value*
Tinetti test				
Balance (/16 points)	+ 0.07 ± 1.91	+ 0.40 ± 2.10	.54	.62
Gait (/10 points)	+ 0.87 ± 1.91	+ 0.48 ± 1.64	.43	.43
Total (/28 points)	+ 0.93 ± 3.14	+ 0.88 ± 2.33	.94	.89
TUG test (seconds)	- 1.14 (− 4.75-3.73)	+ 0.41 (− 3.57-2.41)	.49	.06

P-value* adjusted for sex, Body Mass Index and Mini-Mental-State Examination.

*WBV* = Whole Body Vibration; *TUG* = Timed Up and Go.

### Timed Up and Go test

The WBV group revealed a decrease of the median time of TUG test of - 1.14 (− 4.75-3.73) seconds.

In the control group, this median time increased by + 0.41 (− 3.57-2.41) second. This difference, in favour of the WBV group, was not significant (Table 3). However, when analyses were adjusted for sex, Body Mass Index and Mini Mental State Examination, a positive trend was observed for the WBV group (p=0.06).

### Quantitative walking analysis

Analyses were performed on the 27 patients able to complete the tests without walking assistance during the pre-and the post-tests.

No significant difference was observed between the treated and the control groups for all of the Locometrix^®^ parameters (Table 4).

**Table 4:** Evolution of the Locometrix^®^ parameters for both groups

	**WBV (n = 30)**	**Control (n = 25)**	**P-value**	P-value*
Gait speed (meter/second)				
Simple task	- 0.03 ± 0.21	- 0.05 ± 0.10	.89	.82
Dual task	+ 0.02 ± 0.22	+ 0.05 ± 0.12	.63	.92
P-Value	.10	<.01		
Stride frequency				
(cycle/second)	- 0.22 ± 0.21	- 0.15 ± 0.30	.47	.72
Simple task	- 0.03 ± 0.27	- 0.01 ± 0.21	.87	.31
Dual task	.14	.13		
P-Value				
Stride length (meter)				
Simple task	+ 0.15 ± 0.23	+ 0.06 ± 0.23	.33	.90
Dual task	+ 0.02 ± 0.44	- 0.06 ± 0.21	.78	.41
P-Value	.25	.66		
Stride symmetry (arb. unit.)				
Simple task	+ 4.23 ± 58.6	- 9.21 ± 56.6	.55	.86
Dual task	+ 55.3 ± 88.7	- 11.7 ± 72.1	.06	.47
P-Value	.10	.88		
Stride regularity (arb. unit.)				
Simple task	- 12.2 ± 44.6	- 25.3 ± 72.8	.58	.32
Dual task	- 8.40 ± 47.2	+ 16.7 ± 81.3	.40	.85
P-Value	.81	<.01		
Cranio-caudal mechanic power (Watts/kg)				
Simple task	- 0.05 (− 0.34-0.37)	- 0.09 (− 0.63-0.11)	.65	.83
Dual task	- 0.02 (− 0.21-0.32)	+ 0.01 (− 0.11-0.24)	.87	.87
P-Value	.72	.35		
Antero-posterior mechanic power (Watts/kg)				
Simple task	- 0.07 (− 0.23-0.14)	- 0.02 (− 0.11-0.05)	.81	.82
Dual task	- 0.06 (− 0.18-0.08)	- 0.005 (− 0.7-0.14)	.82	.24
P-Value	.65	.27		
Medio-lateral mechanic power (Watts/kg)				
Simple task	+ 0.02 (− 0.13-0.15)	+ 0.005 (− 0.18-0.10)	.54	.23
Dual task	- 0.07(− 0.11-0.11)	+ 0.06 (− 0.02-0.13)	.34	.64
P-Value	.39	.53		
Counting speed (number/second)				
Simple task	+ 0.15 ± 0.28	+ 0.03 ± 0.11	.19	.54
Dual task	+ 0.08 ± 0.16	+ 0.01± 0.28	.49	.23
P-Value	.34	.78		

P-value* adjusted for sex, Body Mass Index and Mini-Mental-State Examination.

*WBV* = Whole Body Vibration.

### Number of falls

In the WBV group, 17 falls were recorded (mean of 0.55 ± 1.37 falls per patient) compared to 14 falls in the control group (mean of 0.45 ± 1.03 falls per patient) (p= 0.76). The falls were incurred by 6 patients in the WBV group and 7 patients in the control group (p= 0.75) (Table 5).

**Table 5:** - Number of falls recorded for the two groups during the 3-month follow-up

**Parameter**	**WBV (n = 31)**	**Control (n = 31)**	**p-value**
Number of falls recorded during the study	0.55 ± 1.37	0.45 ± 1.03	.76
Number of patients who fell	6 (19.3)	7 (22.6)	.75
History of falls	4 (12.9)	5 (16.1)	.72
No history of falls	2 (6.45)	2 (6.45)	1

*WBV* = Whole Body Vibration.

### Characteristics of responders to Vibrosphere^®^

In the WBV group, 16 patients improved their Tinetti test between pre and post-tests. Those patients, the responders, do not differ from the 14 others except for 3 characteristics: responder patients had a Tinetti gait and a Tinetti balance significantly lower and a median TUG significantly higher than the other patients.

A logistic regression was performed on responder patients (yes/no) to the training for the Tinetti test according to baseline results for these 3 characteristics.

The logistic regression showed that it is not possible, from the baseline results of the Tinetti test and the TUG test to predict the response to the Vibrosphere^®^. Indeed, p-values are > 0.05 for these 3 variables (Table 6).

**Table 6:** Results of the logistic regression of responders to the Vibrosphere^®^ for the Tinetti test

**Parameter**	**Coefficient ± SE**	**p-value**	**OR (unit)**	**CI 95%**
Intercept	9.86 ± 6.96	<.01	1.92	1.18 – 3.12
Tinetti balance baseline (/16)	- 0.02 ± 0.07	.76	0.98	0.85 – 1.12
Tinetti Gait baseline (/12)	- 0.14 ± 0.36	.69	0.87	0.42 – 1.81
Timed Up and Go baseline (seconds)	- 0.75 ± 0.40	.07	0.47	0.21 – 1.08

## Discussion

This randomized controlled trial showed that a 3-month Vibrosphere^®^ training composed of 5 series of 15 seconds of vibrations 3 times a week seems to have no impact on the risk of falls among nursing home residents.

In the literature, three studies have assessed the impact of Whole Body Vibration by means of the Tinetti test [10,12,25]. Unlike the study of Bruyère et al. [12], our study showed no significant improvement in the Tinetti test scores in the WBV group compared to the control group. Bautmans et al. [10] had also noted a significant difference in the Tinetti test scores between the treated and the control patients after 6 weeks of training. However, this difference was due to a decrease of the score of the Tinetti test in the control group and not to an increase in the WBV group. Moreover, we can note that the study of Merkert et al. [25] also showed a significant improvement in the walking parameters for the WBV group. This increase was significantly higher than in our study. Indeed, patients improved their Tinetti test score by 3.9 ± 3.0 points after two weeks of training.

In the present study, the balance parameter was practically unchanged in the WBV group. Given the particularity of the device used in this study, we expected the patients’ balance to improve. However, the Vibrosphere^®^ has not been used optimally during the study because patients held themselves onto wall-bars during the training, which may have decreased the proprioceptive work and thus the expected balance effects of the training.

In addition, we decided to place a cushion under the device to facilitate the training. This cushion might also have decreased the proprioceptive expected work.

Two other hypotheses could explain our results for the Tinetti test. Firstly, the duration of exposure is lower in our study than in others. We exposed our population to 5 series of only 15 seconds of vibrations, which represented 3 minutes 45 seconds of vibrations weekly while all others studies exposed their patients to series of minimum 30 seconds. One study performed sessions of 15 seconds of vibration [11] but the protocol of the training sessions was progressive up to sessions of 60 seconds at the end of the study. Secondly, we can add that the patients’ Tinetti score at inclusion was quite high (mean of 22.9 ± 3.99 points). Therefore, we could not expect a very large increase in the results.

In the literature, seven studies have assessed the impact of Whole Body Vibration by means of the TUG test [10-13,15,16,25]. Our study did not show any improvement in the median time of the TUG test for the WBV group but only a positive trend for the WBV group when analyses were adjusted. Other studies [10,12,13,16] found a significant improvement for the WBV group. In these studies, only the WBV group improved significantly their TUG while patients in the control group did not. Bogaerts et al. [11] showed an improvement of TUG test in both groups of patients. However, the improvement was significantly greater in the treated group. The two last studies [15,25] did not compare the treated group with a control group, which makes the analogy with the present study more difficult. However, these studies demonstrated an improvement of the TUG for the WBV group. It should be noted that in one of this studies, 63% of patients dropped-out in the WBV group [15] and that the other study [25] was a study of only two weeks and had a PEDro score [26] of only 4/10. Given those characteristics, the results of these studies should be interpreted with caution.

Regarding the Locometrix^®^ test, no significant changes of the parameters were observed in our study. Results do not go in the same direction as Pollock et al., [15], who observed a significant increase in the step length and walking speed in the WBV group compared to the control group. Bogaerts et al. [11] and Rees et al. [16] also showed a significant increase in walking speed in treated patients compared to control patients.

Our study presented strengths. Firstly, it is a randomized study, controlled by a group of patients who did not change anything special to their lifestyle. Secondly, this study used a single-blind method to assess the patients’ risk of falls. We can also add that a calculation of statistical power was done. Moreover, we can note that rigorous monitoring of training has been completed.

Despite those characteristics, this study also shows some limitations. The difference between the present results and the results found in the literature could be explained by the various characteristics discussed above but also by the fact that patients enrolled in the treated group might have increased their physical activity just by walking to the training sessions. Therefore, control group patients might have had less physical activity during the training. It also seems important to add that the study was conducted in a population of patients considered as unstable. Indeed, changes in the health of some nursing home patients can be very rapid, sudden, significant but also transient. Therefore, the post-test assessments might have been different if performed on another day. Finally, we can also assume that Vibrosphere^®^ is a device that does not improve the risk of falls in nursing home residents.

## Conclusions

Results of the study showed that 3-month Vibrosphere^®^ training composed of 5 series of 15 seconds of vibrations 3 times a week does not seem to show any benefit on the risk of falls of nursing home residents. Indeed, in these conditions, the Tinetti test, the TUG test and the parameters calculated by the Locometrix^®^ system did not significantly improve in the WBV group compared to the control group.

Further investigation is required to understand the difference observed between our results and literature.

## Abbreviations

WBV: Whole Body Vibration; TUG: Timed Up and Go; Hz: Hertz.

## Competing interest

The authors indicate that they have no competing interests.

## Authors’ contributions

JMC, JYR, OB and CB conceived the project and developed the study hypotheses and the protocol. CB, FB and MM were responsible for data collection, data management and data analyses. MD provided assistance with collecting the study data. CB wrote the drafts of the article under the supervision of OB and DM. All authors have read, reviewed and approved the final manuscript.

## Authors’ information

CB is a Public Health PhD student in the Department of Public Health, Epidemiology and Health Economics, University of Liège, Belgium. DM is a Professor in the Department of Motricity Sciences, University of Liège, Belgium. MM is a Physiotherapy student at the University of Liège, Belgium. FB is a Public Health student at the University of Liège, Belgium. MD is a Physiotherapy PhD student in the Department of Motricity Sciences, University of Liège, Belgium. JMC is chief of the Physical Medicine and of the Physiotherapy Departments of the University of Liège, Belgium. JYR is Head of the Department of Public Health of the University of Liège, Belgium. Finally, OB is a Professor in the Department of Public Health, Epidemiology and Health Economics, University of Liège, Belgium.

## Pre-publication history

The pre-publication history for this paper can be accessed here:

http://www.biomedcentral.com/1471-2318/13/42/prepub
